# Galanin Protects against Nerve Injury after Shear Stress in Primary Cultured Rat Cortical Neurons

**DOI:** 10.1371/journal.pone.0063473

**Published:** 2013-05-14

**Authors:** Meili Liu, Wei Song, Ping Li, Yan Huang, Xianghui Gong, Gang Zhou, Xiaoling Jia, Lisha Zheng, Yubo Fan

**Affiliations:** Key Laboratory for Biomechanics and Mechanobiology of Ministry of Education, Beijing, China, School of Biological Science and Medical Engineering, Beihang University, Beijing, China; University of Iowa, United States of America

## Abstract

The neuropeptide galanin and its receptors (GalR) are found to be up-regulated in brains suffering from nerve injury, but the specific role played by galanin remains unclear. This study aimed to explore the neuroprotective role of galanin after shear stress induced nerve injury in the primary cultured cortical neurons of rats. Our results demonstrated that no significant changes in cell death and viability were found after galanin treatment when subjected to a shear stress of 5 dyn/cm^2^ for 12 h, after increasing magnitude of shear stress to 10 dyn/cm^2^ for 12 h, cell death was significantly increased, while galanin can inhibit the nerve injury induced by shear stress with 10 dyn/cm^2^ for 12 h. Moreover, Gal2-11 (an agonist of GalR2/3) could also effectively inhibit shear stress-induced nerve injury of primary cultured cortical neurons in rats. Although GalR2 is involved in the galanin protection mechanism, there was no GalR3 expression in this system. Moreover, galanin increased the excitatory postsynaptic currents (EPSCs), which can effectively inhibit the physiological effects of shear stress. Galanin was also found to inhibit the activation of p53 and Bax, and further reversed the down regulation of Bcl-2 induced by shear stress. Our results strongly demonstrated that galanin plays a neuroprotective role in injured cortical neurons of rats.

## Introduction

Galanin(Gal) is a widely distributed neuropeptide, with 29–30 amino acids, that regulates various endocrine, pain and cognitive functions, including learning and memory in the central nervous system (CNS) [1], [2]. Galanin is also important in some pathological processes, especially in traumatic brain injury [3], whereby it is up-regulated in the brain following damage to the central nervous system [1], [4]. Galanin mRNA levels are also increased after transient focal cerebral ischemia, galanin acting via GalR1 causes an anti-allodynic effect on neuropathic pain [5], [6]. Most importantly, galanin is significantly elevated in some neurodegenerative diseases, particularly in Alzheimer’s disease [7]. However, the specific role of galanin in nerve injury is still not well understood. If galanin can be shown to block nerve damage, it may be useful in the development of a new treatment strategy for nerve injuries.

High-velocity impacts induce direct high shear stress to the brain, which often caused traumatic brain injury during a traumatic event [8]. Previous studies demonstrated that fluid shear stress can be used to model neural injury in *vitro* [9], [10] The neurobiological mechanisms of the initial cellular damage in nerve injury is crucial [3]. Injured neurons show increased membrane permeability, localized microtubule disruption, organelle accumulation and axonal bead formation [11]. However, the direct relationship between shear stress and neuronal damage is still unknown.

Another viewpoint is that shear stress is good for neuronal regeneration and nerve development [12]. It has been proposed that the mechanical environment is an important factor in nerve regeneration. The mechanical environment plays a central role in the physiology of various tissues. Shear stress affects mechanoreception, such as ion channels and integrin/focal adhesions, cellular response, such as intracellular calcium and nitric oxide production, and cytoskeletal remodeling [11]. Chafik et al. reported that shear stress is an important component of the natural environment for axonal regeneration and enhances cellular adhesion, proliferation, and alignment of Schwann cells [13]. However, the certain effects of shear stress on nerve cells are still unclear. This study aims to explore the impact of shear stress on primary cultured cortical neurons.

In addition, the protective role of galanin in shear stress-induced injury in the cultured cortical neurons of rats will be investigated. There are three subtypes of galanin receptors identified to date (GalR1, GalR2, and GalR3), all of which are G-protein coupled [14], [15]. Previous study have shown that the protective role of galanin is mediated by GalR2 [16], but the subtypes of GalR involved in galanin protection after shear stress injury need to be explored.

Moreover, the application of shear stress to primary cultured cortical neurons activates the pro-apoptosis pathways involving Bax and p53, and inhibits anti-apoptotic Bcl-2. Bax is a pro-apoptotic factor in the signal pathway [17], and a pore-forming cytoplasmic protein, which is considered to promote cell death [18]. In this study, p53-Bax dependent apoptosis pathway will be investigated after shear stress, the ability of galanin to inhibit cell death will be explored.

## Materials and Methods

### Ethics Statement

All experiments involving the use of animals were in compliance with Provisions and General Recommendation of Chinese Experimental Animals Administration Legislation and were approved by Beijing Municipal Science & Technology Commission (Permit Number: SCXK (Beijing) 2006-0008 and SYXK (Beijing) 2006-0025).

### Cell Cultures

Primary cultures of rat cortical neurons were prepared from 1-day-old newborn rats, as described by previous methods [19]. The neurons were seeded on a glass microscope slide at 5×10^5^ cells/ml. Cultures were maintained in modified Eagles medium (DMEM), 10% new born bovine serum, 5% D(+)-glucose, 50 IU/ml penicillin and 0.05 mg/ml streptomycin at 37°C with 95% air and 5% CO_2_. On the second or third day, 4 µg/ml cytosine arabinoside were added to the medium to suppress the proliferation of glia cells. The medium was renewed every 3 days during the culturing period.

### Shear Stress Assay

A parallel-plate flow chamber was used to shear the cultured cortical neurons [20]. After the cells had adhered to the slide, a silicone gasket was sandwiched between the glass slide and an acrylic plate to create a rectangular flow channel. Different magnitudes of shear stress were generated by the flow across the channel resulting from the height difference between two reservoirs. The system was kept at 37°C and equilibrated with 95% humidified air containing 5% CO_2_.

The cultures were subjected to shear impulse levels of 5, 10 and 20 dyn/cm^2^ for 1 h, 4 h, 8 h, 12 h, and 24 h. Any morphological changes to the neurons were recorded (IX 71, Olympus, Japan).

### Drugs and Treatments

Cultured cortical neurons were pre-incubated with galanin (Gal, Tocris, Bristol, UK) at varying concentration from 1 nM to 10 µM for 24 h whilst under shear stress stimulation. Galantide (galanin (1–13)-substance P (5–11), Bachem, King of Prussia, PA), a galanin receptor antagonist (100 nM) was added to medium 24 h before shear stress in vitro. Galanin 2–11 (Galanin receptor 2/3 agonist, HD Biosciences Co. Ltd), were added to the medium 24 h before the initiation of shear stress.

### Measurement of Cell Death: MTT Assay and TUNEL Assay

After galanin treatment, cell viabilities were tested using a 3-(4,5-dimethylthiazole -2-yl)-2,5-diphenyl- tetrazolium bromide (MTT) assay [21], [22] Neuronal cell death was measured using a TdT-mediated biotin-dUTP nicked-end labeling (TUNEL) assay [23], [24], [25]. 4′-6-Diamidino-2-phenylindole (DAPI) was used to stain the nuclei of cultured cortical cells. Cell samples were viewed under a fluorescent microscope using an excitation wavelength of 450–500 nm and detection wavelength of 515–565 nm (green).

### Real Time-PCR Analysis

Galanin receptors were tested using an Real time-PCR analysis to determine whether galanin protection is mediated through its receptors and which subtypes of galanin receptors (GalR) are responsible for protection against nerve injury triggered by shear stress. Cells were harvested and total RNA was isolated with Trizol reagent (Invitrogen, Carlsbad, CA). Total RNA (2 µg) were reversely transcribed using M-MLV Reverse Transcriptase (Invitrogen, Carlsbad, California) and oligo-d(T) 15 random primers (Takara, Shiga, Japan). The real time PCR primer sequences were as follows: GalR1, 5′- AGGCTTACGTGGTGTGCACTTTC-3′ (coding sense) and 5′-GCCATGATATGCCAAATACCACAA-3′(coding antisense).GalR2, 5′- CATCG TGGCGGTGCTTTT-3′ (coding sense) and 5′-AGCGGGAAGCGACCAAAC-3′ (coding antisense). GalR3, 5′- CCTGCCTCAACCCGCTCGTC -3′(coding sense) and 5′-TGAAGGCGGTGGTGGTGGTG-3′ (coding antisense); GAPDH (glycerol dehyde-3- phosphate dehydrogenase) 5′- GGCACAGTCAAGGCTGAGAATG-3′ (coding sense) and 5′-ATGGTGGTGAAGACGCCAGTA -3′ (coding antisense).

The mRNA levels of GalR1, GalR 2, GalR 3 and GAPDH were analyzed by quantitative real-time RT-PCR. A 1 µl cDNA sample was added to 5 nmol of each primer, 10 µl of 2×SYBR Green Supermix (Takara, Kyoto, Japan) and PCR-grade water to a volume of 20 µl. Three replicas were performed in the real-time RT-PCR analysis. Real-time PCR was performed in an iCycler iQ real-time PCR detection system (Bio-Rad). Controls were performed with no reverse transcription or water for each gene to demonstrate the specificity of the primers and the lack of DNA contamination in samples. PCR cycling conditions were as follows: initial 95°C for 30 seconds, then 40 cycles using 95°C for 10 seconds, and 58°C for 35 seconds. Melt curve analysis was performed on the iCycler over the range 55°C to 95°C by monitoring iQ SYBR green fluorescence with increasing temperature (0.5°C increment changes at 10-second intervals). Quantification of the results was done Quantification of the results was done using the comparative CT method [26] and for internal normalization, the housekeeping gene GAPDH was employed. The standard curves were generated by serial dilutions of sample cDNA in five 10-fold dilution steps and used for regression analyses. The final results of real time-PCR were expressed as the ratio of mRNA of control. The data are presented as mean ± SD.

### Patch Clamp Assay

To determine the electrophysiological activity of cells after shear stress and galanin treatment, the cultured cortical neurons were subjected to a patch clamp assay. Whole-cell recording of rat cortical neurons was obtained with the help of a Heka EPC10 amplifier. Recording pipettes were filled with solution containing 122.5 mM Cs-gluconate, 17.5 mM CsCl, 2 mM MgCl_2_, 10 mM HEPES, 0.5 mM EGTA, 4 mM ATP with pH adjusted to 7.2–7.4 by CsOH. The pipette voltage was clamped at −60 mV throughout the whole cell patch recording. EPSCs were recorded for 10 minutes and the results were normalized by the average amplitudes.

After data was collected by Pulse software (HEKA, German), it was normalized by the average amplitude of evoked EPSCs without galanin treatment.

### Immunoprecipitation and Western Blots Assay

Cells were washed in a PBS buffer and lysed in a lysis buffer (Beyotime, China) with 1% phenylmethanesulfonyl fluoride (PMSF). Activated Bax was immunoprecipitated from cell lyastes with 6A7 anti-activated-Bax antibody and precleaned protein-A sepharose beads (Sigma, St. Louis, MI) overnight at 4°C. The beads were collected and washed 3 times in the cell lysis buffer before denaturation and SDS-PAGE.

Samples were fractionated in a 12% SDS-PAGE gel and electrophoretically transferred onto PVDF membranes. The membranes were blocked for 2 hours with 5% non-fat milk in Tris-buffered saline containing 0.05% Tween-20 (TBST) at 37°C, and then incubated with polyclonal antibodies overnight at 4°C; goat anti-Bax antibody (N20,1∶1000), rabbit anti-p53 antibody (1∶500), rabbit anti-Bcl-2 antibody (1∶500) were purchased from Santa Cruz Biotechnology (Santa Cruz, CA). After washing 4×10 min in TBST, the membranes were incubated with an HRP-conjugated anti-goat antibody (1∶3000) and anti-rabbit antibody for 2 hours.

After four washes in TBST, optical density was analyzed under a universal imaging hood 2 (Bio-Rad, USA). The relative density was calculated by the total absolute density of total Bax/GAPDH, Bcl-2/GAPDH, and p53/GAPDH.

### Statistical Analysis

Data are given as means ± S.E.M. For statistical comparison, a t-test or one-way ANOVA followed by Tukey’s test was employed. The following p-values were considered to be statistically significant: * *p*<0.05, **p<0.01 and ***p<0.001.

## Results

### Effect of Shear Stress on the Primary Cultured Cortical Neurons

**Figure 1:**
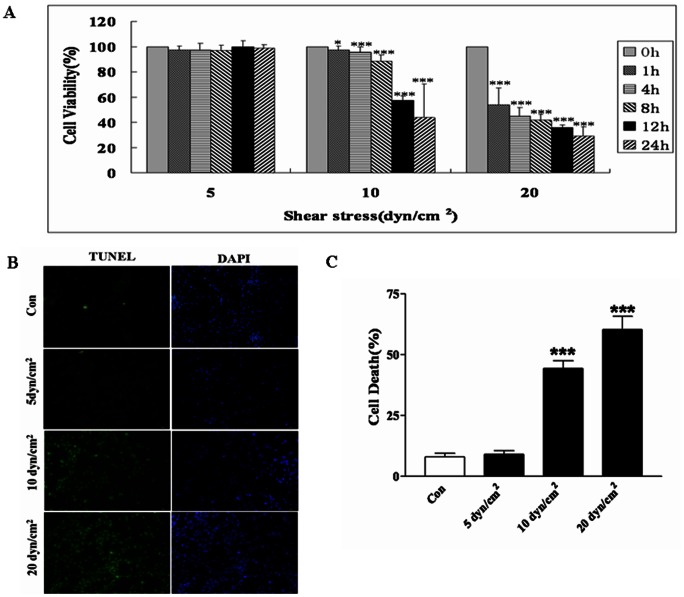
Effect of shear stress on primary cultured cortical neurons. A) Histograms show that the cell viability decreases significantly after shear stress of 5, 10, 20 dyn/cm^2^ for 1 h, 4 h, 8 h, 12 h and 24 h respectively by MTT assay. B) Photomicrographs and C) histograms show that as shear stress levels increased (from 5, 10 dyn/cm^2^ to 20 dyn/cm^2^) TUNEL positive cells increased significantly by TUNEL assay. *p<0.05, **p<0.01 and ***p<0.001 were considered to be statistically significant.

In order to confirm the influence of shear stress on the primary cultured cortical neurons, different levels of shear stress were applied for 0 h,1 h, 4 h, 8 h, 12 h, 24 h respectively in this study (Fig. 1A). Cell viability decreased significantly as the shear stress increased (from 5 dyn/cm^2^, 10 dyn/cm^2^ to 20 dyn/cm^2^). There was no significant change at 5 dyn/cm^2^, while a shear stress of 20 dyn/cm^2^ significantly decreased cell viability up to 30% (n = 10, *p*<0.001), and resulted in serious nerve injury. Starting at 10 dyn/cm^2^ for 0 h,cell viability decreased significantly as shear stress time increased (n = 10, *p*<0.001). Although a large number of cells died, the culture was still about 60% viable at 10 dyn/cm^2^. Therefore, it can be considered a good research model under this condition. The viability was further confirmed by TUNEL assay (Fig. 1B). The number of dead cells increased significantly after shear stress with 10 dyn/cm^2^ and 20 dyn/cm^2^ for 12 h (Fig. 1C), but no significant difference was observed with 5 dyn/cm^2^ for 12 h.

Therefore, our results clearly demonstrated that low shear stress (5 dyn/cm^2^) is not likely to cause cortical neuron injury. Moreover, shear stress of 20 dyn/cm^2^ can seriously disrupt the cell cytoskeleton.

### Effect of Galanin on Injured Cortical Neurons after Shear Stress

**Figure 2:**
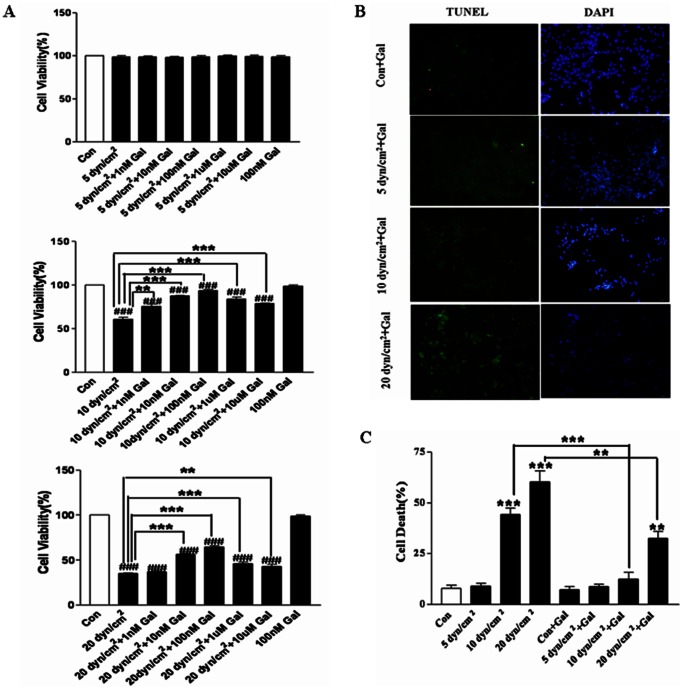
Influence of galanin on shear stress-induced neurotoxicity in primary cultured cortical neurons. A) Histograms show that pretreatment with galanin (Gal) at 1 µM, 100 nM, and 10 nM for 24 h counteracts the shear stress-induced decrease in cell viability, assessed by MTT assay at 10 dyn/cm^2^ and 20 dyn/cm^2^. Cell viability does not change after shear stress of 5 dyn/cm^2^ and there is no marked influence of 100 nM galanin alone on the cell viability. B) Photomicrographs and C) histograms show that pretreatment with 100 nM of galanin for 24 h reduces shear stress-induced nerve injury assessed by TUNEL assay. *p<0.05, **p<0.01 and ***p<0.001 were considered to be statistically significant (Scale bars: B, 50 µm)**.**

To explore the influence of galanin on shear stress-induced nerve injury, the cultured cortical neurons were treated with 1 nM, 10 nM, 100 nM, 1 µM or 10 µM of galanin, or vehicle as a control. After the above pretreatments for 24 h, shear stress of 5 dyn/cm^2^, 10 dyn/cm^2^ and 20 dyn/cm^2^ was applied for 12 h (Fig. 2). As shown in Fig. 2A, there were no significant changes in cell viability after shear stress of 5 dyn/cm^2^ for 12 h. But at 10 dyn/cm^2^, cell death was clearly evident (n = 10, t = 14.64, *p*<0.001). Galanin significantly inhibits shear stress-induced neuronal cell death (Fig. 2A) at the concentrations of 1 nM to 10 µM. No side effects were found with 100 nM galanin treatment (n = 10, t = 0.80, *p* = 0.44). Compared to the shear stress (10 dyn/cm^2^) group, galanin treatment significantly increases cell viability (1 nM, n = 10, t = 2.89, *p*<0.05; 10 nM, n = 10, t = 11.06, *p*<0.001; 100 nM, n = 10, t = 10.82, *p*<0.001). However, at concentrations higher than 100 nM (1 µM, n = 10, t = 5.03, *p*<0.001; 10 µM, n = 10, t = 7.31, *p*<0.001), the protective effects of galanin decrease gradually (Fig. 2A). Shear stress at 20 dyn/cm^2^ for 12 h resulted in widespread cell death (n = 10, t = 108.39, *p*<0.001). However, galanin treatment at 1 nM, 10 nM and 100 nM increased cell viability, but at the concentrations higher than 100 nM, cell viability decreased. Moreover, galanin treatment did not improve cell viability after shear stress at 20 dyn/cm^2^ for 12 h (cell viability remained about 30%).

Cell death was further examined using a TUNEL assay (Fig. 2B and 2C). Our study showed that shear stress of 5 dyn/cm^2^ for 12 h will not result in nerve injury, indicating that galanin treatment did not play role in this group. While shear stress of 10 dyn/cm^2^ for 12 h induced a marked increase in cell death (n = 5, t = 9.83, p<0.001), galanin at 100 nM resulted in a marked decrease in positive TUNEL cells (Fig. 2B). Galanin treatment reduced cell death by about 31.85% (n = 5, t = 7.35, *p*<0.001) (Fig. 2C). However, at 20 dyn/cm^2^ for 12 h (n = 5, t = 4.01, *p*<0.05), galanin did not reduce overall cell death,the TUNEL positive cells still appeared after galanin treatment, and cell death remained about 32.65% (Fig. 2C).

### Involvement of Galanin Receptor (GalR) in the Protective Effects of Galanin against Shear Stress - Induced Nerve Injury

**Figure 3:**
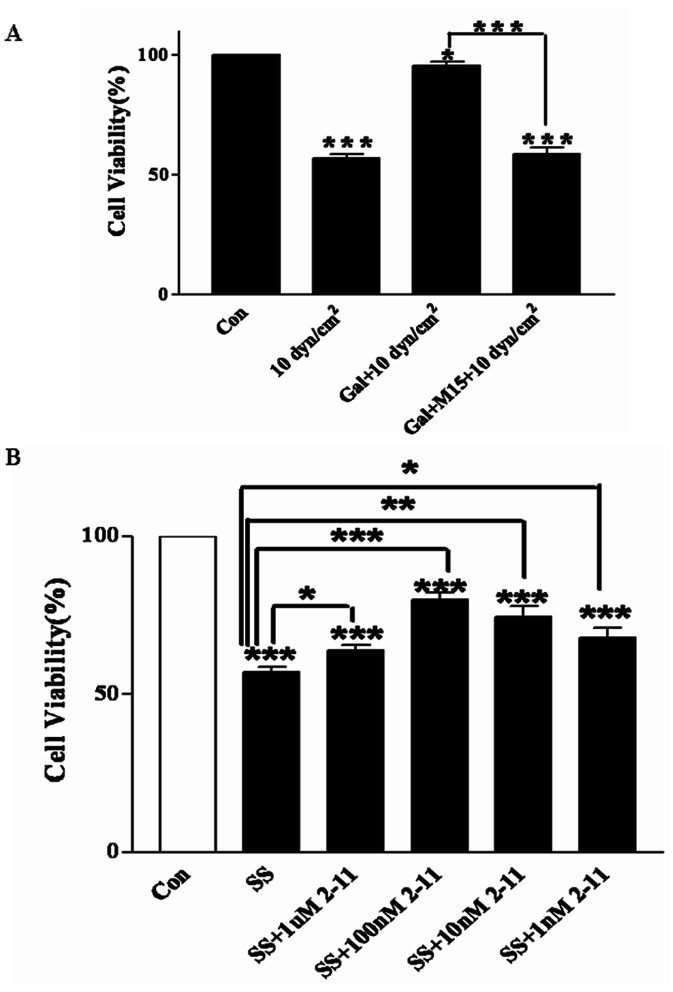
Involvement of GalR2 in the protective effects of galanin against shear stress-induced neurotoxicity. A) Galantide (M15), a galanin receptor antagonist, blocks the protective effects of galanin after shear stress of 10 dyn/cm^2^ for 12 h. B) Gal2-11, a galanin receptor 2/3 agonist, and pretreatments of 1 µM, 100 nM, 10 nM of the galanin receptor 2/3 antagonist Gal2-11 show significant protective effects against shear stress-induced nerve injury tested by MTT assay. *p<0.05, **p<0.01 and ***p<0.001 were considered to be statistically significant.

Three types of galanin receptors (GalR1, GalR2, and GalR3) were checked in the primary cultured cortical neurons of rats, as showed in Fig. 3. Both GalR1 and GalR2 were found, however, GalR3 was not expressed in our system.

The presumed protective effects of galanin receptors against shear stress-induced nerve injury were also examined, as shown in Fig. 3A. Compared to the Galanin plus shear stress treatment group, galanin-induced protection was significantly inhibited by galantide (*n* = 6; *t* = 12.07, *p<*0.001), tested using an MTT assay, suggesting the involvement of galanin receptors. As galantide is a non-specific antagonist to the three types of galanin receptors, an agonist (Gal2-11) specific to GalR2 and GalR3 was used. As shown in Fig. 3B, compared to the shear stress treatment group (SS), pretreatment with 1 µM (*n* = 6, t = 2.49, *p<*0.05), 100 nM (*n* = 6, t = 7.27, *p<*0.001), 10 nM (*n* = 6, t = 3.97, *p<*0.01) and 1 nM (*n* = 6, t = 3.08, *p<*0.05) of Gal2-11 demonstrated significant protective effects against shear stress-induced nerve injury. The results suggested that GalR2 may be involved in the protective effects of galanin in primary cultured cortical neurons.

### Effect of Shear Stress on the Expression of Galanin Receptors

**Figure 4:**
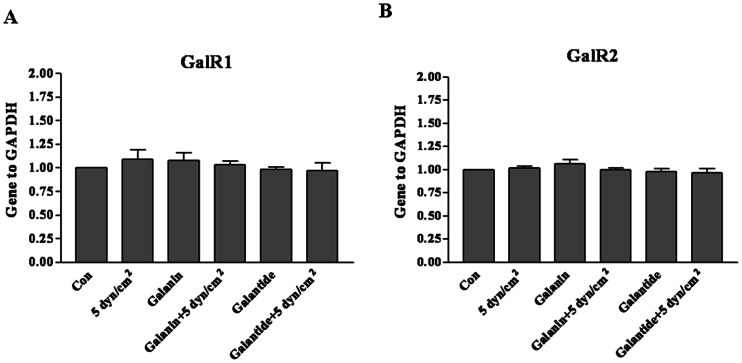
Expressions of mRNAs for GalR1, R2 and R3 after shear stress of 5 dyn/cm^2^ for 12 h. Quantitative real time PCR results show that GalR1 (Fig. 4A)and GalR2 (Fig. 4B) did not change significantly, and there was no GalR3 expression in the cortical neurons with galanin or galantide treatment after shear stress of 5 dyn/cm^2^ for 12 h. The data are presented as mean ± SD (n = 3).

Low shear stress of 5 dyn/cm^2^ did not significantly influence cell death. Neither did the galanin treatment (100 nM) on the expression level of galanin receptors GalR1(Fig. 4A) and GalR2(Fig. 4B) after low shear stress, while there is no expression of GalR3 in our system in quantitative real-time RT-PCR assay(Fig. 4).

**Figure 5:**
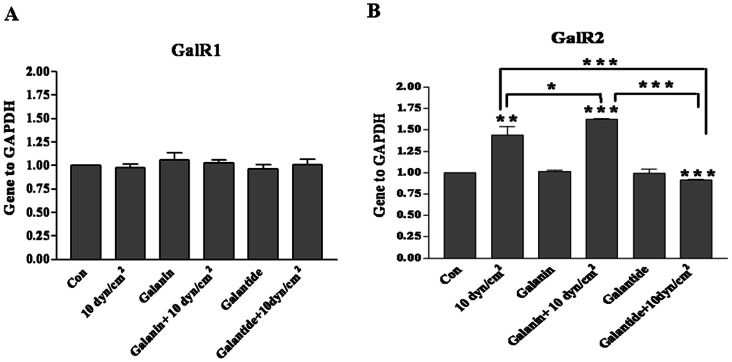
Expressions of mRNAs for GalR1, R2 and R3 after shear stress of 10 dyn/cm^2^ for 12 h. (A) GalR1 mRNA levels in cultured cortical neurons after shear stress of 10 dyn/cm^2^ for 12 h with galanin or galantide treatment was assessed by real time PCR assay. (B) GalR2 mRNA levels in cultured cortical neurons after shear stress of 10 dyn/cm^2^ for 12 h with galanin or galantide treatment was assessed by real time PCR assay. GalR3 is not expressed in our system. The data are presented as mean ± SD (n = 3). *p<0.05, **p<0.01 and ***p<0.001 were considered to be statistically significant.

After high shear stress stimulation (10 dyn/cm^2^ for 12 h), galanin or galantide treatment did not influence GalR1 expression in quantitative real-time RT-PCR assay (Fig. 5A). Our quantitative real-time RT-PCR assay results revealed that cells exposed to high shear stress stimulation expressed GalR2(Fig. 5B) at a higher level than low shear stimulation (n = 3, *p*<0.01). Galanin can promote the expression of GalR2 (n = 3, *p*<0.05) after high shear stress stimulation (Fig. 5B). In addition, galantide at 100 nM can effectively inhibit the expression of GalR2 after high shear stress stimulation (n = 3, *p*<0.001), which can also effectively block the galanin role after high shear stress stimulation (n = 3, *p*<0.001), there is no expression of GalR3 after high shear stress stimulation, results showed in Fig. 5.

Thus, 10 dyn/cm^2^ shear stress for 12 h may induce marked cell death and significantly increase GalR2 expression. Galanin treatment can effectively protect neuronal injury, which is mediated by GalR2.

### Effect of Galanin on Excitatory Postsynaptic Currents (EPSCs)

**Figure 6:**
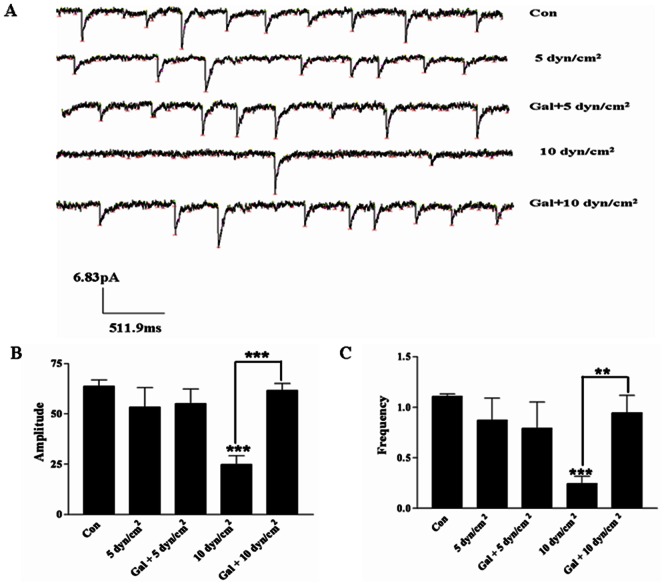
Effect of galanin on the excitatory postsynaptic currents (EPSCs) after shear stress. A) EPSC of cultured cortical neurons after shear stress of 5 dyn/cm^2^ and 10 dyn/cm^2^ by patch clamp assay. B) Shear stress of 5 dyn/cm^2^ for 12 h can not cause a significant change in amplitude. But shear stress of 10 dyn/cm^2^ for 12 h cause significantly decrease the amplitude, and pretreatment of galanin cause a significant increase in amplitude. C) Frequency of cortical neurons after galanin treatment. Shear stress of 5 dyn/cm^2^ for 12 h can not cause a marked decrease in frequency, but shear stress of 10 dyn/cm^2^ for 12 h induced a significant decrease in frequency, while galanin increased the frequency of cortical neurons after shear stress with 10 dyn/cm^2^. *p<0.05, **p<0.01 and ***p<0.001 were considered to be statistically significant.

To explore the potential protective effects of galanin, the EPSCs were recorded after shear stress of 5 dyn/cm^2^ and 10 dyn/cm^2^ for 12 h in primary cultured cortical neurons using a patch clamp assay (Fig. 6A). Our results showed that 5 dyn/cm^2^ shear stress could not induce significant changes in amplitude of EPSCs after galanin treatment (n = 4, t = 0. 20, p = 0.85). The amplitude decreased significantly after 10 dyn/cm^2^ shear stress (n = 4, t = 8.70, *p*<0.001), while galanin treatment could effectively increase the amplitude at 10 dyn/cm^2^ for 12 h (n = 4, t = 5.69, *p*<0.01) (Fig. 6B).

We also found that the frequency of EPSCs follows the same pattern under both shear stress levels. However, galanin could effectively inhibit the effect of 10 dyn/cm^2^ shear stress (n = 4, t = 3.25, *p*<0.05) (Fig. 6C).

### Galanin Inhibits Cell Apoptosis by p53-Bax -Bcl2 Signal Pathway

**Figure 7:**
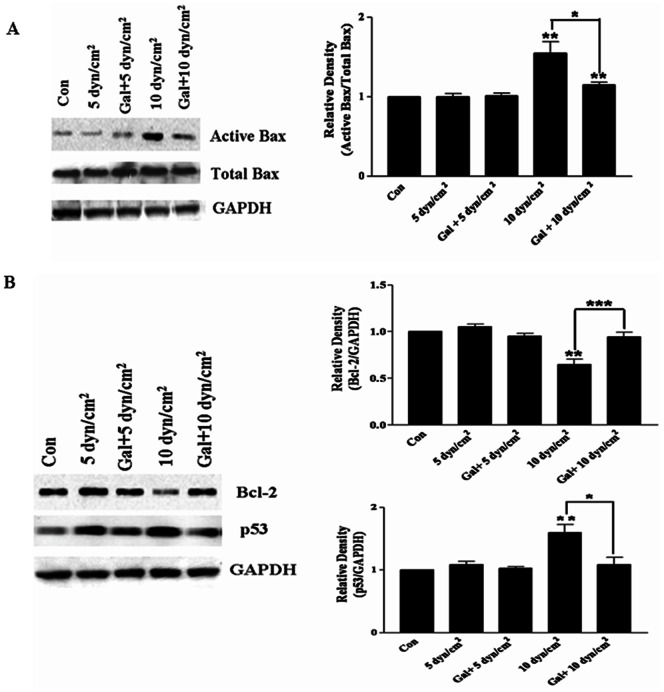
Effect of galanin on the cell apoptosis in the primary cortical neurons. A) The effect of galanin on Bax in cortical neurons after shear stress was assessed by immunoprecipitation assay. There was no significant decrease in Bax after shear stress of 5 dyn/cm^2^, but there was a significant increase in active Bax after shear stress of 10 dyn/cm^2^; galanin inhibits the increase of active Bax. B) The expression of Bcl-2 and p53 were tested by galanin treatment. Shear stress of 10 dyn/cm^2^ for 12 h decreased Bcl-2 and increased p53 expression, while galanin may inhibit the effect of shear stress. There were no significant changes in p53 and Bcl-2 after shear stress of 5 dyn/cm^2^ for 12 h. *p<0.05, **p<0.01 and ***p<0.001 were considered to be statistically significant.

One of the common pro-apoptotic factors up-regulated in all nerve injury is Bax [22]. Its potential galanin protection mechanism against cell death was demonstrated. In this study, our immuoprecipitation assay showed that shear stress of 5 dyn/cm^2^ for 12 h could not significantly increase the expression of Bax (n = 6, t = 0.04, *p* = 0.96), meanwhile, galanin could not perform appropriately at this level of shear stress (n = 6, t = 0.33, *p* = 0.75). 10 dyn/cm^2^ for 12 h significantly increased total Bax and activated Bax in rat neurons (n = 6, t = 3.77, *p*<0.01). In addition, galanin inhibited Bax expression (n = 6, t = 3.24, *p*<0.05), and could inhibit activated Bax as well if normalized to total Bax levels (right panel of Fig. 7A).

To determine the degree of galanin protection, Bcl-2 and p53 were measured using a western blots assay (Fig. 7B). Shear stress of 5 dyn/cm^2^ for 12 h could not change the expression of Bcl-2 and p53, but 10 dyn/cm^2^ induced a significant decrease in Bcl-2 (n = 4, t = 6.15, *p*<0.01) and an increase in p53 (n = 4, t = 4.44, *p*<0.01). After galanin treatment, p53 decreased significantly (n = 4, t = 2.19, *p*<0.05) and Bcl-2 was significantly up-regulated (n = 4, t = 17.66, *p*<0.001) (Fig. 7B). This suggested that galanin may protect against cell death under a shear stress of 10 dyn/cm^2^ for 12 h.

## Discussion

Previous studies have reported that galanin is up-regulated following central nerve injury [2], [27], and importantly, it is significantly elevated in some neurodegenerative diseases [2]. However, whether galanin induces cell death or protects against cell death is still not well understood.

Neurons can also be damaged through mechanical loading, which can cause pain and loss of function [9], [28]. Kilinc’s study indicated that 45 dyn/cm^2^ with 20 ms onset time can induce fluid shear stress injury (FSSI) in cultured primary chick forebrain neurons [9]. Previous studies have focused on related injury models [8], [29], but the associated cellular pathways are still not clearly understood. Thus, this study set out to determine the effect of shear stress on primary cultured cortical neurons. Interestingly, it was found that low shear stress of 5 dyn/cm^2^ did not cause nerve injury, neuronal cell viability did not decrease, and TUNEL positive cells were not evident.

The effect of shear stress on the cellular response mechanisms to damage in primary cortical neurons was investigated. At 10 dyn/cm^2^, cells did not detach and TUNEL positive cells increased significantly, but neuronal damage was apparent. While shear stress of 20 dyn/cm^2^ caused marked nerve injury, obvious cell death. This study opted for 10 dyn/cm^2^ for 12 h because of the high degree of nerve injury seen with greater shear stress, which is consistent with previous studies [10].

Interestingly, galanin did not play a significant role under a low shear stress of 5 dyn/cm^2^, when cells were not injured. However, 10 dyn/cm^2^ or 20 dyn/cm^2^ for 12 h both induced marked cell death. At the same time, galanin can effectively inhibit nerve injury and increase cell viability in an MTT and TUNEL assay under high shear. But, 20 dyn/cm^2^ for 12 h was too high and resulted in cell damage; galanin could not improve cell viability, as the damage was mostly related to cell detachment under high shear.

Galanin concentrations showing protective effects *in vitro* ranged from 1 nM to 10 µM with maximal effects at 100 nM, which is consistent with previous reports [7]. In addition, data from the MTT assay and TUNEL assay confirmed these neuroprotective effects.

To determine which receptor subtypes are mainly responsible for the protective mechanism, the presence of GalR1, GalR2, and GalR3 was examined after shear stress. Three galanin receptors GalR1, GalR2 and GalR3 are believed to mediate the neuroprotective effects [14]. GalR1 and GalR2 are widely expressed in the central nervous system, but GalR3 is more limited [30]. In this study, GalR1 and GalR2 were easily detected while GalR3 remained undetectable. To identify which receptor mediates protection, Gal2-11, a GalR2 and GalR3 agonist, was used. Since GalR3 was undetectable, while galanin treatment can not induce significant changes in GalR1 in an real time RT-PCR assay, but GalR2 changed significantly, so our results suggest that only GalR2 is involved in protection, this is consistent with previous studies [16], [31].

Assessing the electrophysiological characters of neurons after galanin treatment, it was found that low shear stress of 5 dyn/cm^2^ for 12 h did not change the EPSCs in a patch clamp assay. The shear stress remained low enough to prevent nerve injury and maintain normal electrophysiological characters. Here, shear stress of 10 dyn/cm^2^ for 12 h induced marked nerve injury, resulting in a significant decrease in EPSC. However, galanin significantly increased the amplitude and frequency of EPSC, improved the plasticity of cortical neurons, and helped to recover the electrophysiological function of neurons after shear stress.

Since one of the common pro-apoptotic factors up-regulated in all insults examined in the present study is Bax [18], the involvement of Bax regulation was examined as a possible protection mechanism. Bax is a cytoplasmic protein, which is considered to promote cell death [22]. In this study, the level of active Bax/total Bax increased significantly in the cultured cortical neurons after shear stress of 10 dyn/cm^2^ for 12 h in an immunoprecipitation assay, while galanin down-regulated Bax expression. However, there were no significant changes in Bax after shear stress of 5 dyn/cm^2^ for 12 h. This demonstrated that galanin does not have an influential role after low shear stress. Our results showed that activated Bax is significantly elevated in primary cultured cortical neurons exposed to shear stress of 10 dyn/cm^2^ for 12 h, and pretreatment with galanin prevented Bax activation. It is possible that galanin exerts neuroprotective effects through the Bax apoptosis pathway. Studies have demonstrated changes in the expression of apoptosis related proteins in neurodegenerative diseases, such as p53, Bcl-2, and Bax [32], [33], [34]. Upon apoptosis, Bax undergoes a conformational change which exposes its hydrophobic C terminal, and then it is translocated to mitochondria, leading to the release of cytochrome c and the activation of caspase [35]. Galanin was found to inhibit shear stress–induced Bax activation. p53 can mediate Bcl-2 inhibition, thereby modulating Bax activity to facilitate the release of cytochrome c [36] and p53 can also activate Bax directly [37]. Moreover, p53 is known to modulate Bcl-2 family gene expression [38]. Our data showed that galanin inhibited p53 expression in primary cultured cortical neurons. Interestingly, expression of Bcl-2 markedly decreased after shear stress of 10 dyn/cm^2^ for 12 h, but galanin may act to inhibit this. The above results suggest that galanin exerts its effects through the p53-Bax dependent apoptosis pathway after shear stress of 10 dyn/cm^2^ for 12 h.

### Conclusion

Low shear stress of 5 dyn/cm^2^ can not induce marked cell death, but as magnitude of shear stress increased to 10 dyn/cm^2^, cell death are more significantly. Galanin inhibits cell death by down-regulating Bax and p53. Our data strongly suggests that galanin protects cortical neurons against shear stress-induced injury, it may provide a new therapeutic intervention for nerve injury.
